# Assessing Neurocognition via Gamified Experimental Logic: A Novel Approach to Simultaneous Acquisition of Multiple ERPs

**DOI:** 10.3389/fnins.2016.00001

**Published:** 2016-01-29

**Authors:** Ajay K. Nair, Arun Sasidharan, John P. John, Seema Mehrotra, Bindu M. Kutty

**Affiliations:** ^1^Department of Neurophysiology, National Institute of Mental Health and Neuro SciencesBengaluru, India; ^2^Multimodal Brain Image Analysis Laboratory, National Institute of Mental Health and Neuro SciencesBengaluru, India; ^3^Department of Psychiatry, National Institute of Mental Health and Neuro SciencesBengaluru, India; ^4^Department of Clinical Neurosciences, National Institute of Mental Health and Neuro SciencesBengaluru, India; ^5^Department of Clinical Psychology, National Institute of Mental Health and Neuro SciencesBengaluru, India

**Keywords:** paradigm design, cognitive functions, decision making, simultaneous ERPs, P300

## Abstract

The present study describes the development of a neurocognitive paradigm: “Assessing Neurocognition via Gamified Experimental Logic” (ANGEL), for performing the parametric evaluation of multiple neurocognitive functions simultaneously. ANGEL employs an audiovisual sensory motor design for the acquisition of multiple event related potentials (ERPs)—the C1, P50, MMN, N1, N170, P2, N2pc, LRP, P300, and ERN. The ANGEL paradigm allows assessment of 10 neurocognitive variables over the course of three “game” levels of increasing complexity ranging from simple passive observation to complex discrimination and response in the presence of multiple distractors. The paradigm allows assessment of several levels of rapid decision making: speeded up response vs. response-inhibition; responses to easy vs. difficult tasks; responses based on gestalt perception of clear vs. ambiguous stimuli; and finally, responses with set shifting during challenging tasks. The paradigm has been tested using 18 healthy participants from both sexes and the possibilities of varied data analyses have been presented in this paper. The ANGEL approach provides an ecologically valid assessment (as compared to existing tools) that quickly yields a very rich dataset and helps to assess multiple ERPs that can be studied extensively to assess cognitive functions in health and disease conditions.

## Introduction

Neurocognitive studies employ experimental manipulation of variables such as attention, perception, memory, and decision making to elicit details with regard to cortical information processing. Event Related Potentials (ERPs) are electrophysiological correlates of cognitive processing. There are many well established cognitive task paradigms available in the literature for evaluating the cognitive functions in human subjects. One of the most widely used tasks is the oddball paradigm using either visual or auditory stimuli.

A wide variety of ERPs have been studied extensively–for example: the C1, an early visual component that occurs within few milliseconds following the stimulus presentation and not much influenced by the visuospatial attention (Kelly et al., 2008), P50 an early auditory component used for assessment of sensory gating (Smith et al., 2013), MMN—an index of sound discrimination even in the absence of attention(Chen et al., 2014), N1-P2 complex for corollary discharge mechanism—which discriminates sensations generated by one's own action and those generated externally (Wang et al., 2014), N170—a component that marks rapid perception of faces and familiar patterns (Caharel et al., 2013), N2pc—a component that reflects focus of attention on a potential target during visual search (Cespón et al., 2013), LRP—a component that indicates cortical readiness to motor response (Vainio et al., 2014), P300—an indicator of cognitive discrimination based on sustained attention and memory mechanisms (Shaikh et al., 2013), and ERN—a component that reflects error detection (Arbel and Donchin, 2014) etc.

However, most of the existing methodological approaches (including oddball tasks) provide experimental stimulus manipulations to elicit one or two ERPs at a time. Studying multiple variables one at a time does not provide details of interaction effects among neurocognitive variables and hence provides only little data on overall neurocognitive processing capabilities of the brain. Secondly, if the effect size is small, many trials are needed to be carried out to ensure sufficient statistical power. In reality, we encounter multi-sensory information simultaneously and the brain is capable of carrying out multiple ways of information processing. Newer innovative techniques are hence warranted to make the tasks more realistic and ecologically valid. An innovative approach, “Manipulation of Orthogonal Neural Systems Together in Electrophysiological Recordings” (MONSTER) developed by Kappenman and Luck (2012) introduced the concept of studying multiple ERPs (four) by stimulating orthogonal neural systems. However, the problems associated with lack of ecological validity still remains with the procedure.

The ANGEL (“Assessing Neurocognition via Gamified Experimental Logic”) approach has been designed to overcome these limitations and has been designed by gamifying the MONSTER framework while building a logical experimental framework for dissociating multiple ERPs simultaneously. Gamification is the use of aspects of game design in non-game contexts (Deterding et al., 2011). The ANGEL paradigm uses the visual oddball paradigm as its base and employs a number of game elements to make the task engaging and allow the study of decision making in a variety of contexts. ANGEL simultaneously employs multiple audio and visual stimuli so that participants engage in decision making in the presence of multiple distractors. This is more representative of real life situations and therefore more ecologically valid than the approaches taken by cognitive assessments that minimize the number of stimuli and explicitly focus on the variable to be assessed.

The ANGEL paradigm allows assessment of 10 neurocognitive variables, as it is possible to elicit the following ERPs: C1, P50, MMN, N1, N170, P2, N2pc, LRP, P300, and ERN. The paradigm design provides the flexibility to ascertain the main and interaction effects of the variables using over 500 conditions. ANGEL employs an audiovisual sensory motor design and as the approach involves three game levels of increasing complexity, the subjects are motivated to carry out the task efficiently. Using automated scripts, it is possible to selectively analyze the variables of interest while reducing analysis time and minimizing potential researcher bias.

In this paper, we discuss the details of ANGEL design as well as the rationale behind it. We also present sample results from a proof of concept study to demonstrate the validity of the approach. The details of possible data analyses are also provided to highlight various aspects of cognitive information processing. This approach can be used to provide information with regard to dysfunctional brain mechanisms associated with mental/cognitive disorders, understand the cognitive reserve capacities of normal human brain as well as following mind training conditions such as meditation. Additionally, the approach is useful in outcome studies following cognitive remediation training and other interventions.

The design goals of the ANGEL paradigm were: (1) allow simultaneous acquisition of multiple ERPs; (2) provide opportunity to study how these cognitive measures vary with different levels of complexity and indeed how they interact with each other; (3) reuse of a common paradigm (stimulus presentation as well as analysis methods) across a large variety of target populations and study if and how they differ in terms of the cognitive measures; (4) keep the task simple yet engaging for the participant, and (5) minimize the literacy and language requirements for participation.

## Materials and methods

### Participants

A total of 18 participants from both sexes (8 females and 10 males) between 27 and 59 years of age took part in the study. All participants were right handed, non-smokers, and provided written informed consent as approved by Institute Human Ethics Committee (NIMH/DO/SUB-COMMITTEE/2011/Sl.No.1, Basic Sciences) and in accordance with the Declaration of Helsinki (1964). Fourteen participants were healthy and without any medication and four were under stable medication for hypertension (*n* = 3) and for diabetes (*n* = 1). Financial compensation was not provided to the participants. Pre-menopausal female participants were in the follicular phase (within 1 week after menstruation) at the time of the study. Participants had refrained from caffeinated beverages for at least 4 h prior to the recording. All acquisitions were done between 2 and 6 p.m.

### EEG acquisition and data processing

All recordings were conducted in a sound attenuated cabin in the human cognitive research laboratory of the department of Neurophysiology, NIMHANS, with ambient temperature maintained at 25°C. Humidity was not controlled and ranged between 40 and 60%.

During the session, the participants sat comfortably in a chair with armrest in front of a 34 × 27 cm LCD monitor set 90 cm away from the participant. They responded by pressing the first two buttons of the four choice Response Pad (Electrical Geodesics Inc., Eugene, OR, USA) with left or right index fingers. Localized visual instructions were presented as per the participants' language preference. The participants were informed about the rules of ANGEL task before the EEG acquisition for cognitive task performance. In addition, participants were provided with visual instructions as well as a practice session at the beginning of each game level.

EEG was acquired using 128 channel HydroCel Geodesic Sensor Nets connected to the 128 channel Geodesic EEG System 300 acquisition system with Net Amps 300 amplifier and Net Station 4.5.7 software (Electrical Geodesics, Inc., Eugene, OR, USA). Before placing the Sensor Net, scalp preparation was done by gentle scrubbing with cotton dipped in the Potassium Chloride and shampoo solution. EEG was digitized with a resolution of 24 bits with a sampling rate of 1 KHz and corresponding anti-aliasing filter of 500 Hz. No notch filters were used. Impedance for all electrodes was maintained at less than 50 KΩ as recommended by the vendor. Eprime 2.0 stimulus presentation software (Psychology Software Tools, Inc., Sharpsburg, PA, USA) was used for presenting the audio-visual stimuli.

### ANGEL paradigm design

The ANGEL paradigm is a gamified adaptation of the visual oddball paradigm. The task is carried out in three levels with increasing complexity: “Learn and Do” (*Learn*), “Whodunnit” (*Who*), and “Discern and Decide” (*Discern*). Each level has 448 trials (16 blocks each comprising of 25 stimulus trials and 3 baseline trials) and takes about 15 min to complete.

#### Trial details

**Figure 1:**
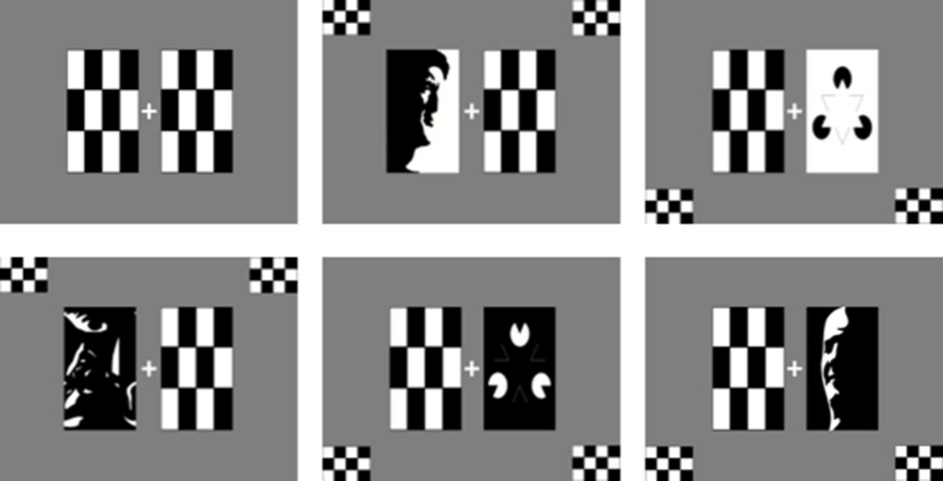
**ANGEL stimulus types**. Clockwise from top left—Checkerboard mask, Mooney face white background, Kanizsa triangle white background, distorted image of Mooney face, distorted image of Kanizsa triangle, and Mooney face on dark background.

**Figure 2:**
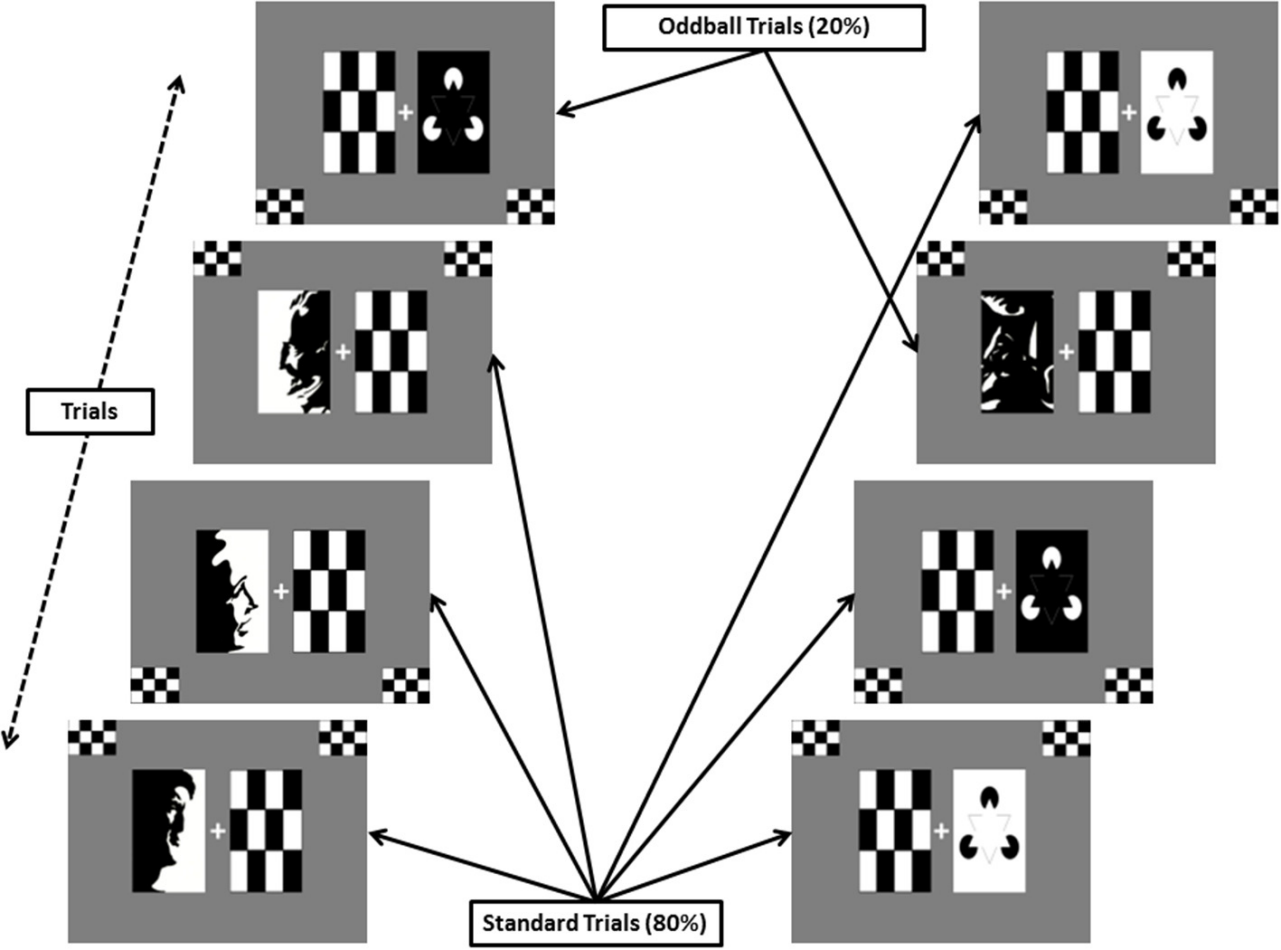
**ANGEL trial blocks**. The block of trials on the left has Mooney face on left side of the fixation sign as standard image type. The block of trials on the right has Kanizsa triangle on right of the fixation sign as standard image type.

Before the trial starts, a white plus sign (+, for fixation) appears in the center of the screen against a gray background along with black and white checkerboards on either side of the plus sign. During the trial, one of the checkerboards is briefly replaced (240 ms duration) in a pseudorandom manner, with one of four types of salient black and white visual stimuli: a Mooney face (Mooney and Ferguson, 1951) or a distorted version of it (randomly picked from a set of 30 each); a Kanizsa triangle (Kanizsa, 1979) or a distorted image with the same components as the Kanizsa triangle (randomly picked from a set of two each) as shown in Figure 1. In each block, a different version of the oddball paradigm is used by presenting the salient image types as follows: One of the four salient image types is presented on one side of the fixation sign for 80% of the time (*Frequent* image category) while two other image types are presented on the other side for a total 20% of the time (10% for each type, forming the *Rare* category). Figure 2. shows two examples of blocks with different stimulus types.

**Figure 3:**
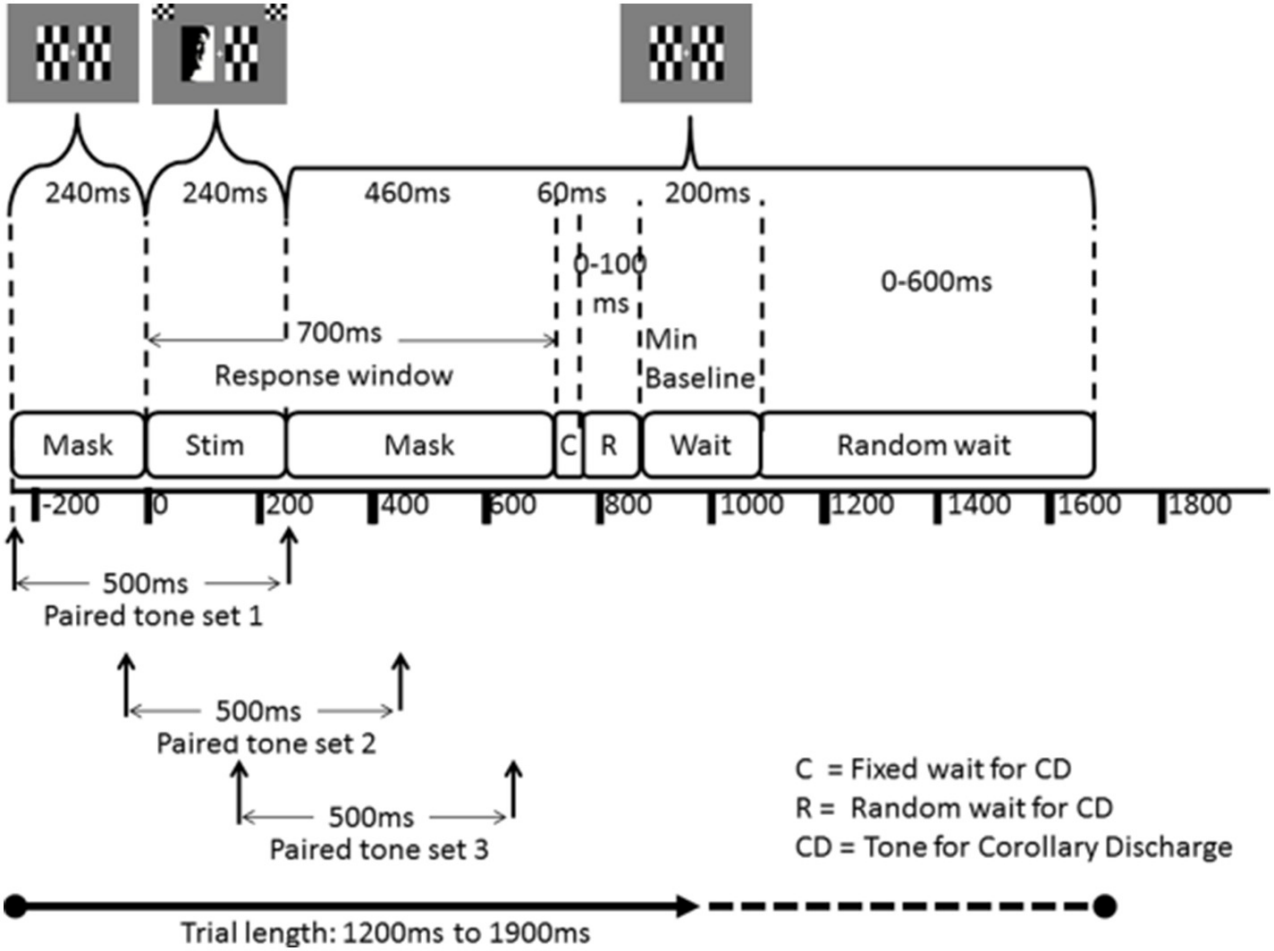
**Structure of an ANGEL trial**. The visual stimulus (Stim) has a pre-stimulus mask (where paired tone stimuli may begin) and a post stimulus mask during which the subject may respond. The tone for Corollary Discharge (CD) may be presented (C) as soon as the subject responds or after a random interval (R) at the end of the response window timeout. Only one of the three paired tone sets are presented in a trial. Every paired-tone trial is followed by three trials with just a blank sound clip. There is a fixed minimum baseline (with mask) of 200 ms at the end of each trial which may be randomly extended by up to an additional 700 ms. Average trial length is 1550 ms (ranging from 1200 to 1900 ms).

Additionally, each trial is presented with multiple audio-visual distractors as detailed in Figure 3. Auditory tones (75 dB) are delivered using earphones with disposable ear plugs. *Standard* auditory tones (80% trials, 1000 Hz, 15 ms long, 500 ms apart) and *Deviant* auditory tones (10% trials, 1500 Hz, 15 ms long) are presented in each block. The auditory tones are presented in one of three sets timed with reference to the visual stimulus onset: 240 ms before, 40 ms before, or 160 ms after, the start of the visual oddball trial. In addition, small checkerboards appear bilaterally on top or bottom (randomly allocated) for different trials as visual distracters as can be seen in Figure 1.

Each trial is 1550 ms long on average (varied between 1200 and 1900 ms). Participants have up to 700 ms to respond (using the index fingers of both hands) to the stimuli after which it is considered a missed response.

In each block, there are 25 active trials followed by three baseline trials [where only the fixation sign (+ sign) is presented on a gray background, without any checkerboard masks].

The average luminous intensity was tested to be similar across blocks using the SHINE Toolbox (Willenbockel et al., 2010) and MATLAB Image processing toolbox. The Mooney face images and their distorted versions were kindly provided by Peter Uhlhaas (Uhlhaas et al., 2006). We prepared the Kanizsa triangles and their distorted versions using the open source graphics editor Inkscape (http://www.inkscape.org/).

#### Game level details

During *Learn*, for half the alternate blocks (eight, odd numbered blocks), participants are asked to passively fixate on the centrally located plus sign without any button press response associated with stimulus presentation while mentally identifying which side of the fixation sign the salient stimuli appear during a trial. However, for the alternate even numbered blocks, participants have to respond to the presentation of the salient stimuli by a button press on the corresponding side. They are also asked to stay focused on the task and ignore all auditory and visual distractors.

During *Who*, participants have to provide a button response during all blocks. However, in this level, for half of the pseudo-randomly allocated blocks, whenever the subject makes a button response, a brief polysyllabic tone (230 ms long) is immediately generated by the system while for the remaining blocks, the tone is generated after the stimulus presentation with a randomly introduced delay. For these latter blocks, if the subject does not make a response, the tone is generated after the timeout period.

During *Discern*, participants are instructed to indicate the salient image type as “meaningful” by pressing the left button or “ambiguous” by pressing the right button. Halfway through the level, following completion of eight blocks, the participants are instructed about rule reversal that they need to press the right button for meaningful images and left button for ambiguous images.

After every two blocks, participants are provided with a feedback on their performance (accuracy and speed). If the accuracy falls below 85%, the feedback text shows “Well tried! Try to respond more accurately.” If the accuracy is in the range of 85–95%, the feedback is “Good job, Keep it up!” For accuracy levels greater than 95% the feedback given is “Outstanding! Now try to respond a bit faster.”

The investigator also has the facility to monitor the subject's response accuracy for each trial and the opportunity to pause the game during the feedback presentations in order to clarify anything or answer any questions.

### Data analysis

The acquired raw EEG data were run through the following processing pipeline. A 0.1 Hz first order high pass filter was run using Net Station 4.5.7 and then the files were exported in Net Station Simple Binary epoch marked format. The events were separately exported as ASCII files. Further ERP preprocessing and analysis was done using custom scripts using EEGLAB v13.4.4b (Delorme and Makeig, 2004)—an open source toolbox and MATLAB version R2013a (MathWorks Inc., Natick, MA, USA).

In brief, the Net Station simple binary files were imported into EEGLAB “.set” format and the channel locations were set as per the 129 channel file supplied by EGI. For ease of discussion, the channel locations compatible to the 10–20 system were renamed while the rest of the channels retain the EGI nomenclature. Events were imported after handling the jitter for sound marker latencies and inserting “S2” sound events 500 ms after “S1” events (these are related to the P50. This step is needed as both the sounds are presented together as one sound clip to avoid timing delays during stimulus presentation). A 40 Hz low pass filter was applied and then artifact correction and removal was done for bad channels, eye blinks, and movement artifacts using the artifact subspace reconstruction (ASR) method implemented in the clean_artifacts function of the clean_rawdata plugin of EEGLAB. We used a threshold of 5 standard deviations for the artifact correction. Independent Component Analysis (ICA) was performed on the cleaned data (down-sampled to 500 Hz and epoched from –0.5 to 0.8 s) using the “runica” algorithm and the ICA weight matrices were saved.

Separately, on the cleaned data files, the removed bad channels were interpolated, data were then down-sampled to 250 Hz and re-referenced to average. Peri-stimulus epoching was done from −1 to 2 s. The saved ICA weight matrices were copied onto the corresponding epoched files. An EEGLAB STUDY was created for C1, P50, MMN, N170, N2pc, CD, LRP, ERN, and P300 in order to evaluate condition differences for each of the following channel measures: ERP, Power Spectra, Event Related Spectral Perturbations (ERSP), and Inter Trial Coherence (ITC).

Statistical significance was set at *p* < 0.05 tested using paired *t*-test (One-way repeated measures ANOVA for N170) with 2000 permutations and False Discovery Rate (FDR) correction for multiple comparisons.

Clustering of Independent Components for source analysis was done using the Measure Projection Toolbox (MPT; an EEGLAB plugin) which uses mutual information similarity between component sources for each measure and identifies spatial domains where these values are consistently found (Bigdely-Shamlo et al., 2013). Statistical significance level was set at *p* < 0.01 with 2000 permutations (default setting). The Measure Projection Analysis (MPA) was done for each of the measures—ERP, Power Spectra, ERSP, and ITC and the anatomical information for each domain was obtained.

ERPLAB v4.0.3.1 (Lopez-Calderon and Luck, 2014), an EEGLAB plugin, was used for visualization of ERP waveforms and comparison of ERPs across multiple game levels.

## Results

**Figure 4:**
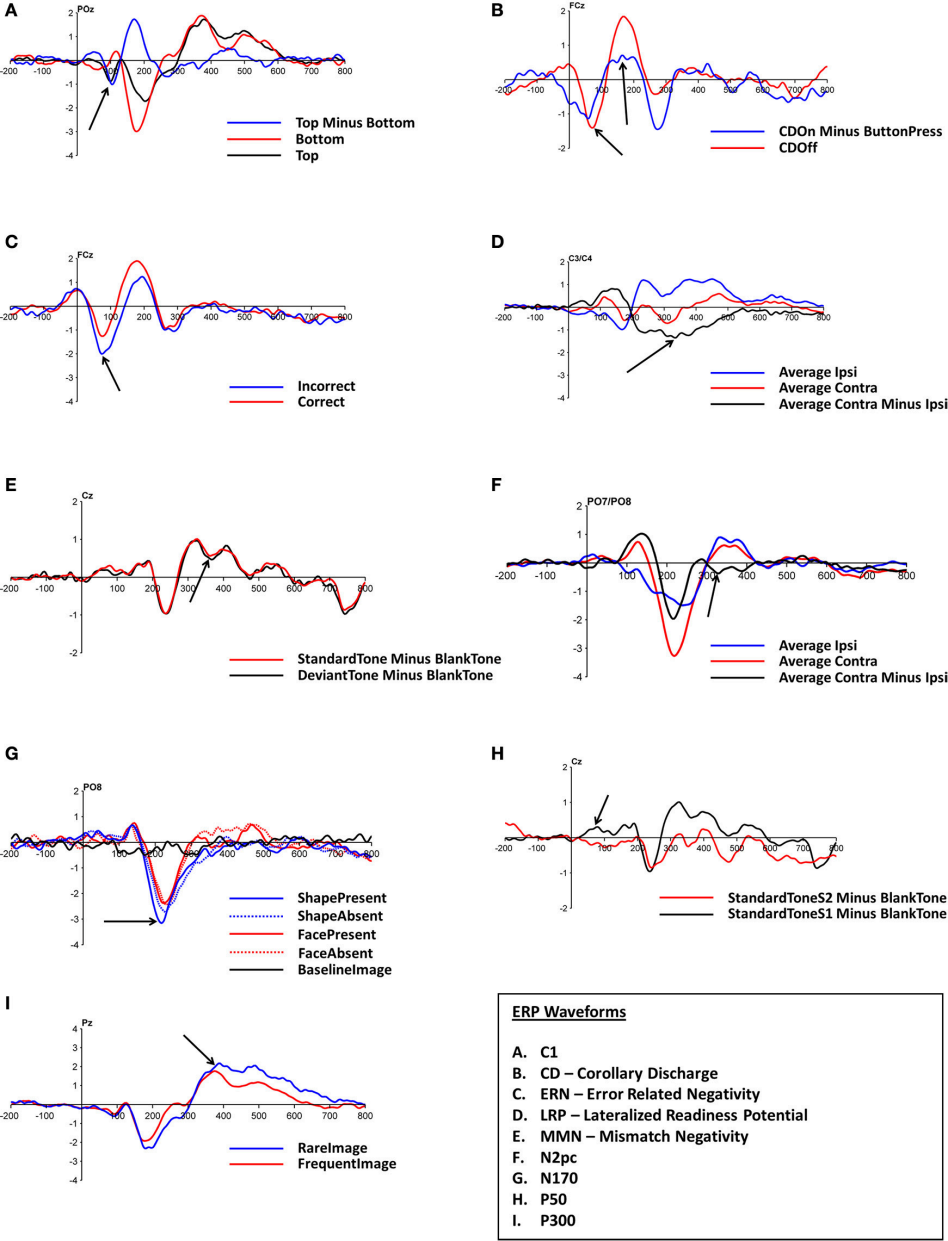
**ERP waveforms for (A) C1-P1 complex for level ***Who***, (B) Corollary Discharge (N1-P2) complex for level ***Who***, (C) ERN (Error Related Negativity) for level ***Discern***, (D) LRP for level ***Who***, (E) MMN (Mismatch Negativity) collapsed across all levels, (F) N2pc for level ***Who***, (G) N170 for level ***Discern***, (H) P50 collapsed across all levels, (I) P300 for level ***Who*****. Arrows indicate the region of interest for the specific ERP.

Figure 4 shows the ERP waveforms of nine neurocognitive measures elicited by ANGEL in the set of healthy participants recruited for the proof of concept study. Regions of interest for each ERP are indicated by arrows. In addition to the conventional ERPs, the study demonstrates the details of other measures such as the corollary discharge mechanism. While it is not possible to fully provide all possible results within journal length limitations, we present a subset of results in order to demonstrate the validity of the ANGEL paradigm using a sample of the variety of analyses that can be performed.

### Event related potential waveforms

**Figure 5:**
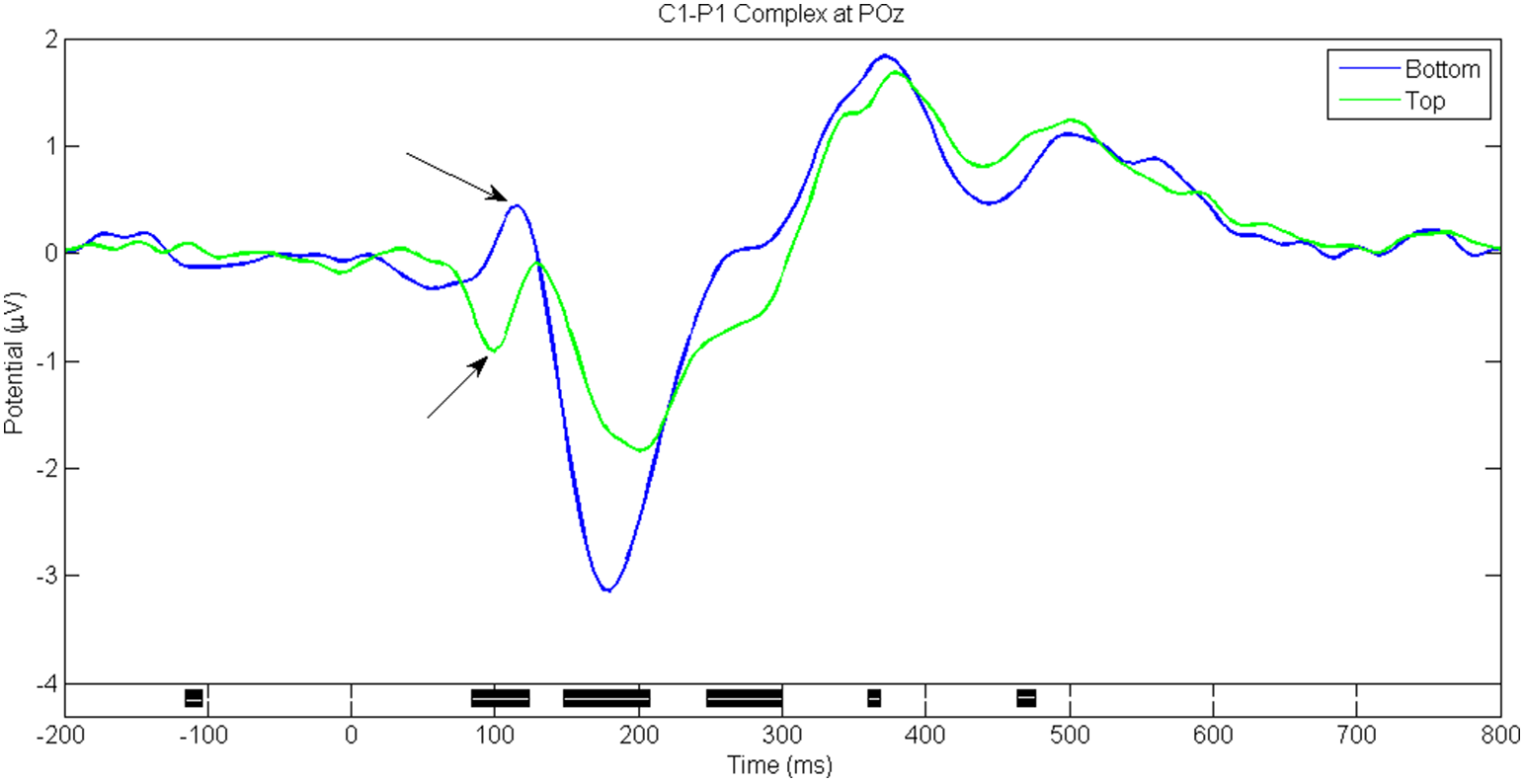
**C1-P1 Complex for game level ***Who*****. Seen at electrode POz around 100 ms post stimulus. Note the opposite polarities seen for Top and Bottom stimuli. Black bars in the bottom panel indicate regions of significant difference between conditions (*p* < 0.05, Paired *t*-test with 2000 permutations with FDR correction).

**Figure 6:**
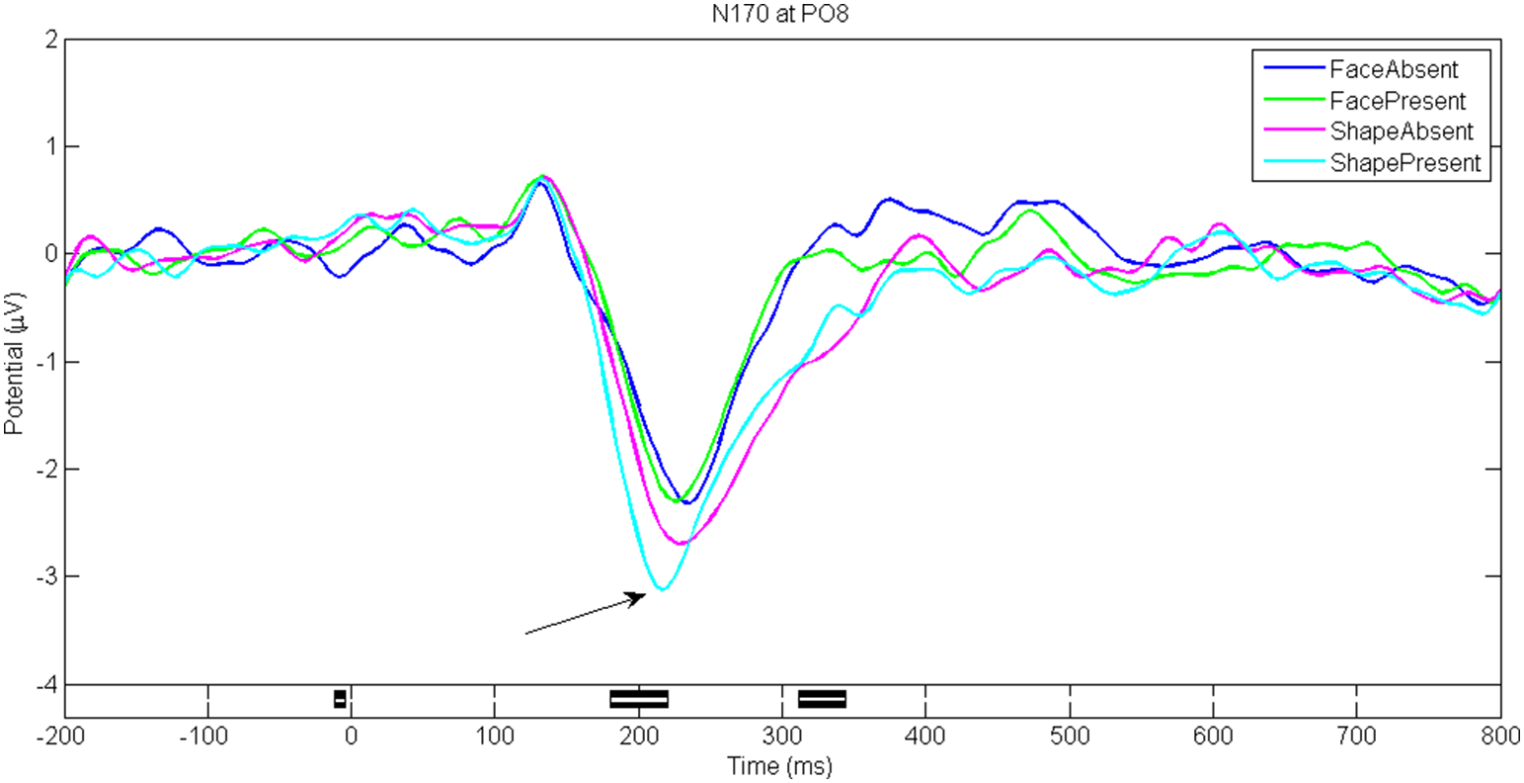
**N170 for game level ***Discern*****. Seen at electrode PO8 between 200 and 250 ms post stimulus. Black bars in the bottom panel indicate regions of significant difference between conditions (*p* < 0.05, Repeated measures One-way ANOVA with 2000 permutations with FDR correction).

**Figure 7:**
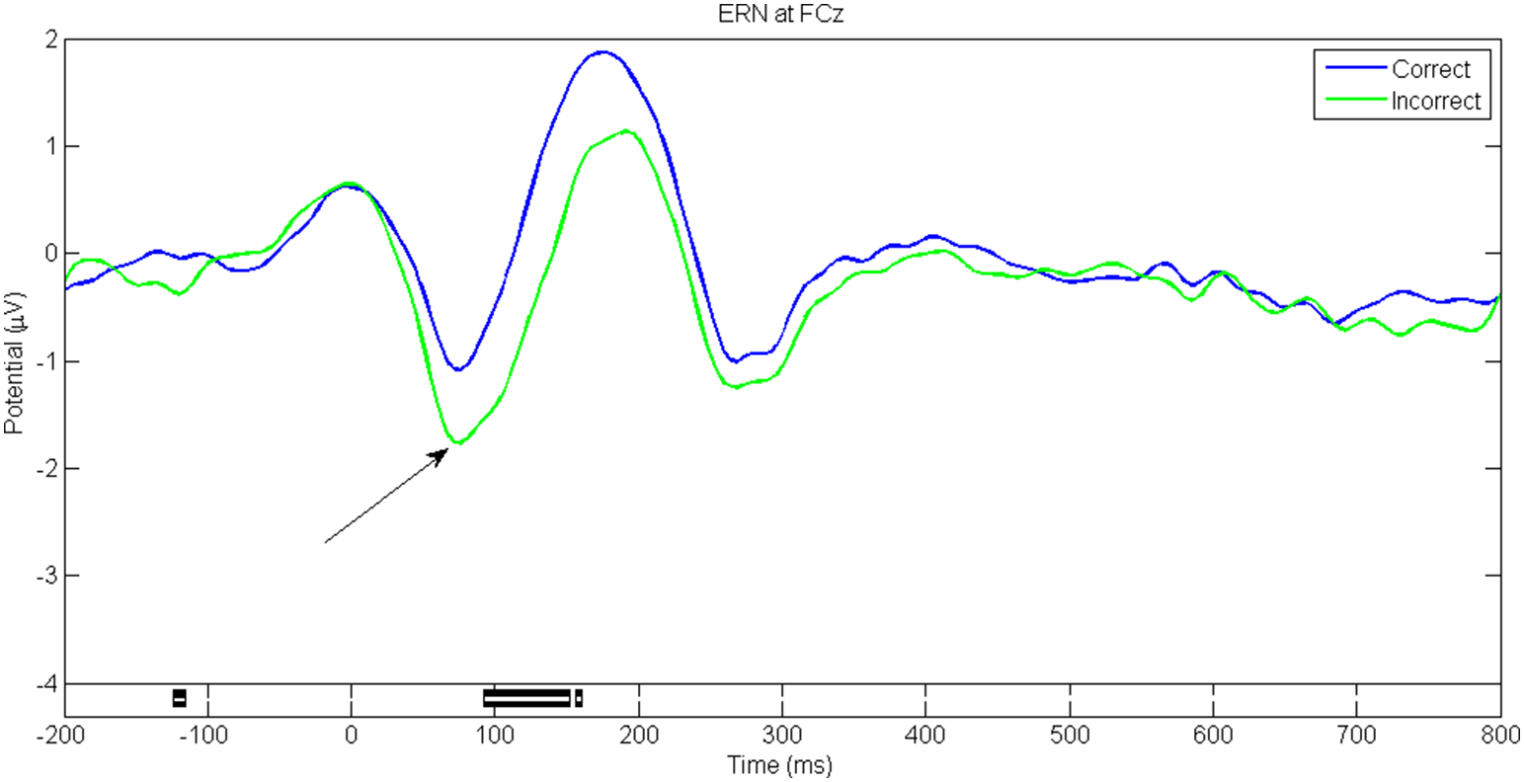
**ERN for game level Discern**. This is response locked and seen at electrode FCz as a negative deflection about 100 ms post response followed by a positive going potential. The negative dip for the correct responses indicates ongoing conflict whereas the larger negative dip indicates the error related negativity. Black bars in the bottom panel indicate regions of significant difference between conditions (*p* < 0.05, Paired *t*-test with 2000 permutations with FDR correction).

**Figure 8:**
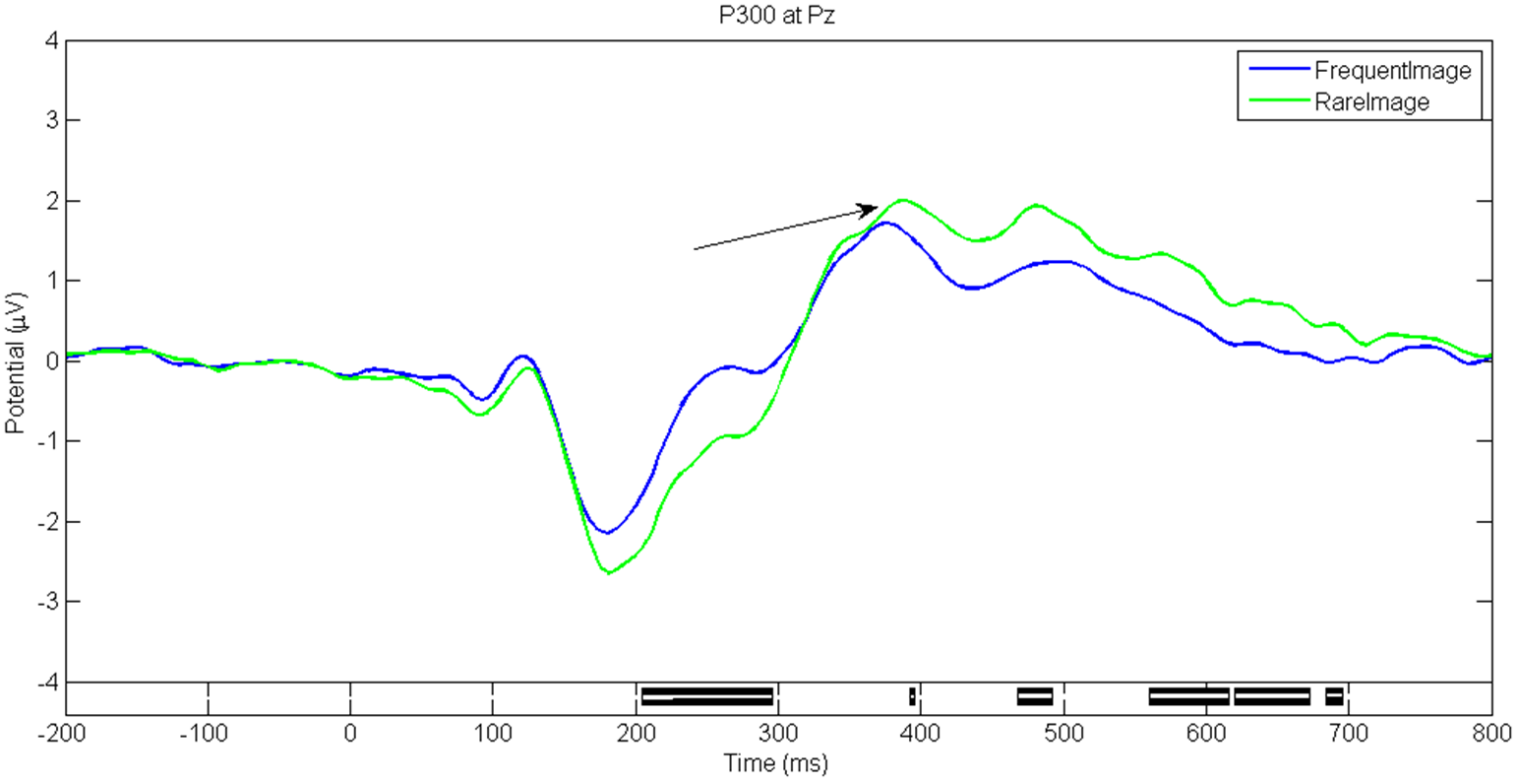
**P300 for game level ***Who*****. Seen at electrode Pz. Rare stimuli evoke a larger P300 peak as compared to frequent stimuli. Black bars in the bottom panel indicate regions of significant difference between conditions (*p* < 0.05, Paired *t*-test with 2000 permutations with FDR correction).

Figures 5–**8** show the ERP waveforms at different latencies obtained using the ANGEL approach: the early (C1), mid (N170), and late latency ERPs (P300) that are stimulus locked and an ERP that is response locked (ERN). The black bars at the bottom show the regions with significant differences between the conditions (*p* < 0.05, using robust measure of paired *t*-test or One-way repeated measures ANOVA as applicable; computed with 2000 permutations and FDR corrected for multiple comparisons).

The C1-P1 complex ranges from 40 to 120 ms. Figure 5 shows the typical C1-P1 waveforms at the electrode POz for the game level *Who*. The distractor stimuli presented bilaterally at the top of the screen have a negative going peak while the distractors at the bottom of the screen show a positive peak that merges with the P1 component.

The N170 waveforms (with a negative going dip between 170 and 250 ms) at electrode PO8 for four different stimuli types (*FacePresent, FaceAbsent, ShapePresent*, and *ShapeAbsent*) for game level *Discern* are shown in Figure 6. Note that the N170 is more prominent for Shapes (Kanizsa triangles and their distorted versions) than for Faces (Mooney faces and their distorted versions). Also note that the N170 waveform in our study has its greatest negative peak close to 220 ms rather than at 170 ms.

The ERN waveforms (with a negative dip before 100 ms followed by a positive peak) at electrode FCz for the trials with *Correct* and *Incorrect* responses for the game level *Discern* are shown in Figure 7. These epochs are response locked unlike the other ERPs. Note that the dip is greater for *Incorrect* trials.

The P300 waveforms (large positive peak around 400 ms) at electrode Pz for trials with *Rare* and *Frequent* stimuli for the game level *Who* are shown in Figure 8. The *Rare* stimuli elicit a larger peak.

### Impact of task complexity

**Figure 9:**
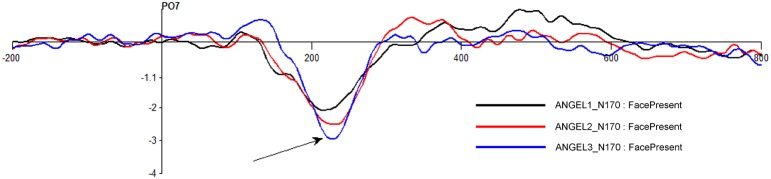
**Effect of task complexity for N170 at electrode PO7 for ***FacePresent*** stimuli across ANGEL game levels ***Learn, Who***, and ***Discern*****.

The effect of task complexity across the game levels for N170 *FacePresent* trials is shown in Figure 9. The N170 amplitude increases with complexity with ANGEL level 1 (*Learn*) having the lowest amplitude (i.e., smallest dip) and ANGEL level 3 (*Discern*) having the maximal amplitude.

### Event related spectral perturbations

**Figure 10:**
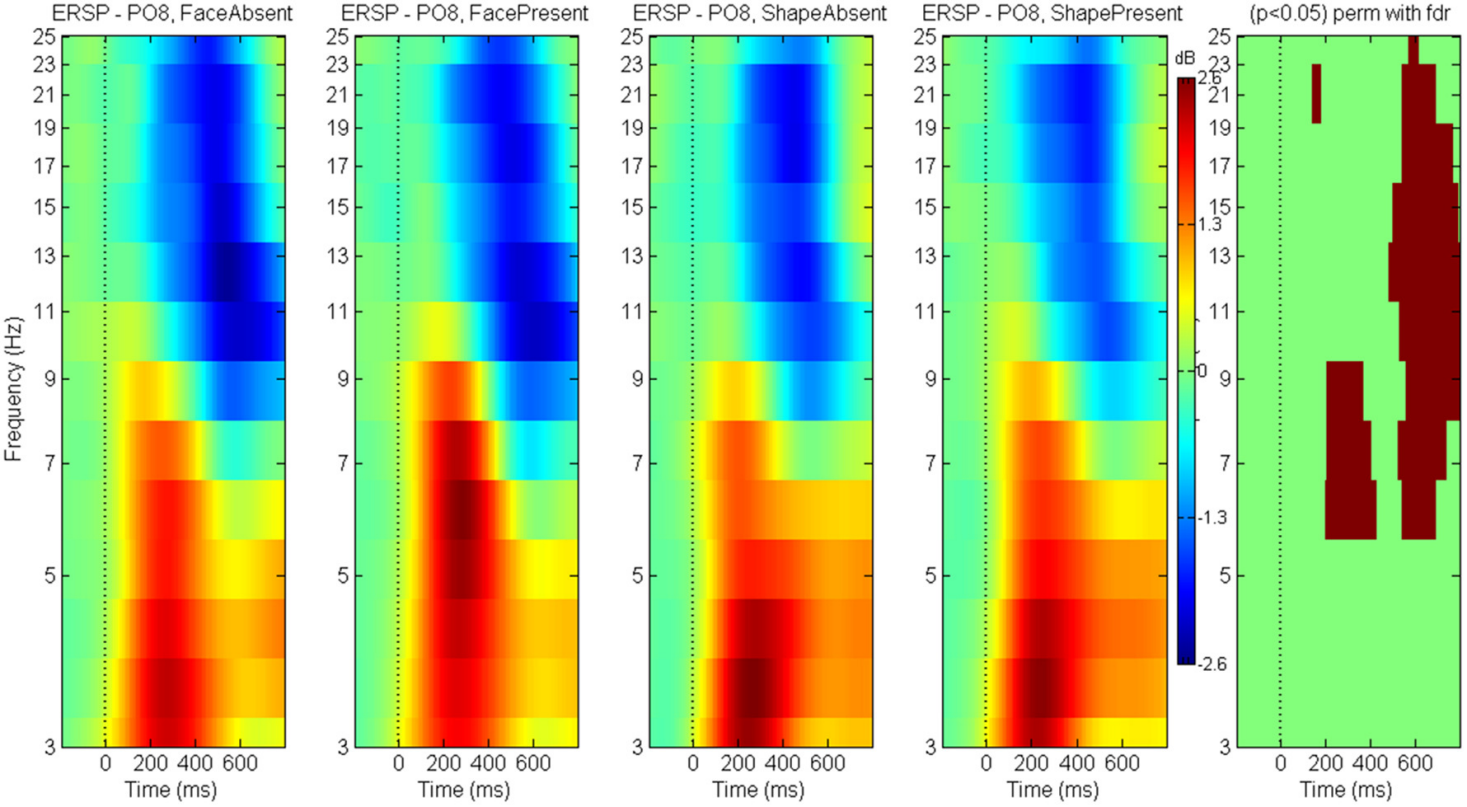
**ERSP for N170**. Event Related Spectral Perturbations for N170 at electrode PO8 for game level *Discern*. Panel on the right indicates the regions with significant differences across conditions (*p* < 0.05 repeated measures One-way ANOVA with 2000 permutations and FDR correction).

**Figure 11:**
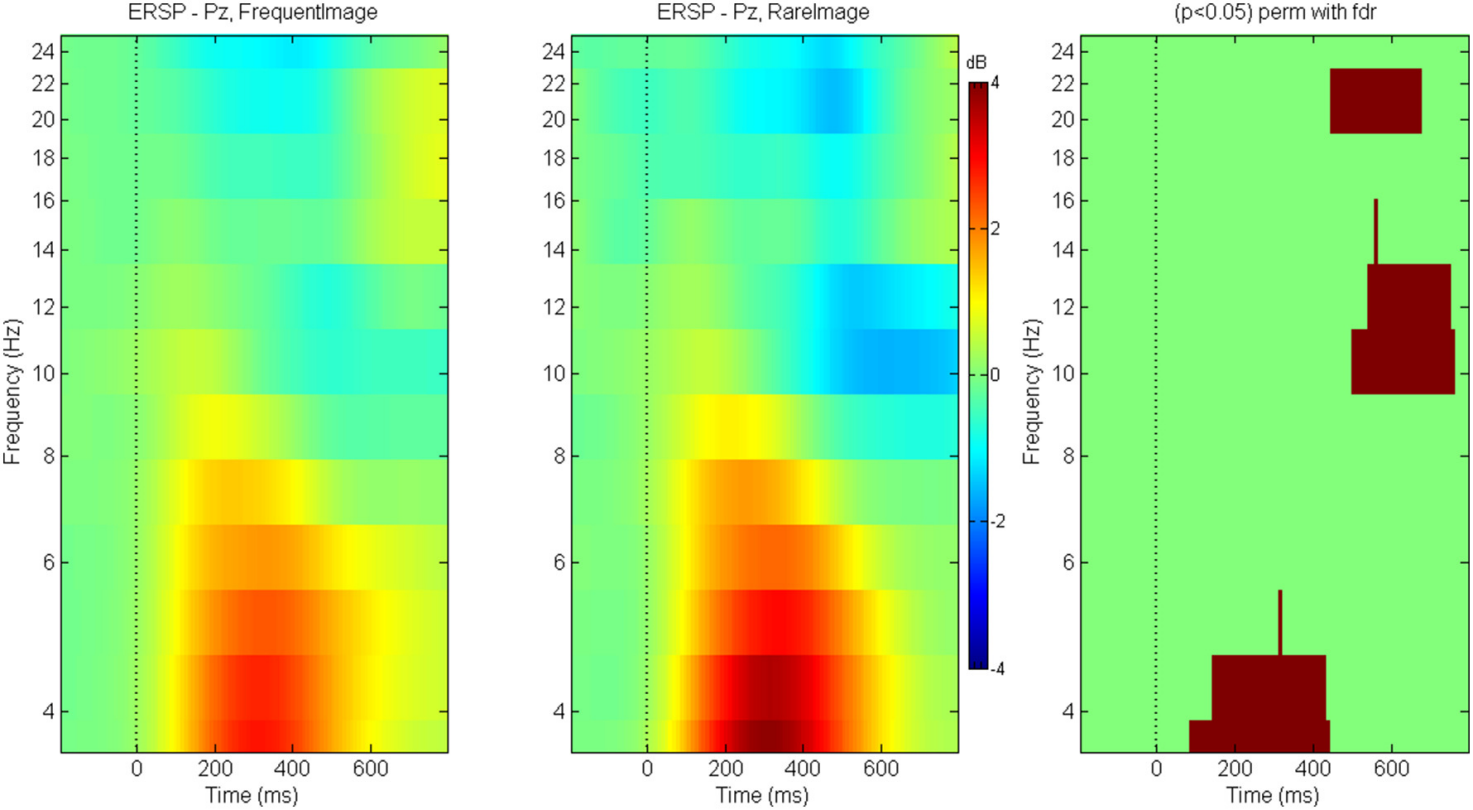
**ERSP for P300**. Event Related Spectral Perturbations for P300 at electrode Pz for game level *Who*. Panel on the right indicates the regions with significant differences across conditions (*p* < 0.05 Paired *t*-test with 2000 permutations and FDR correction).

Between-condition differences in the ERSPs are shown for N170 (Figure 10) and P300 (Figure 11) components. The brown colored areas on the rightmost panel show the regions with significant differences between the conditions (*p* < 0.05, using robust measure of paired *t*-test or One-way repeated measures ANOVA as applicable; computed with 2000 permutations and FDR correction for multiple comparisons).

The N170 between-condition differences for ERSP show greater theta-alpha power for *FacePresent* trials followed by suppression across a wide frequency band (higher theta to beta). The P300 between-condition ERSP differences show delta-lower theta enhancement for *rare* stimuli followed by localized alpha and beta suppression.

### Inter-trial coherence

**Figure 12:**
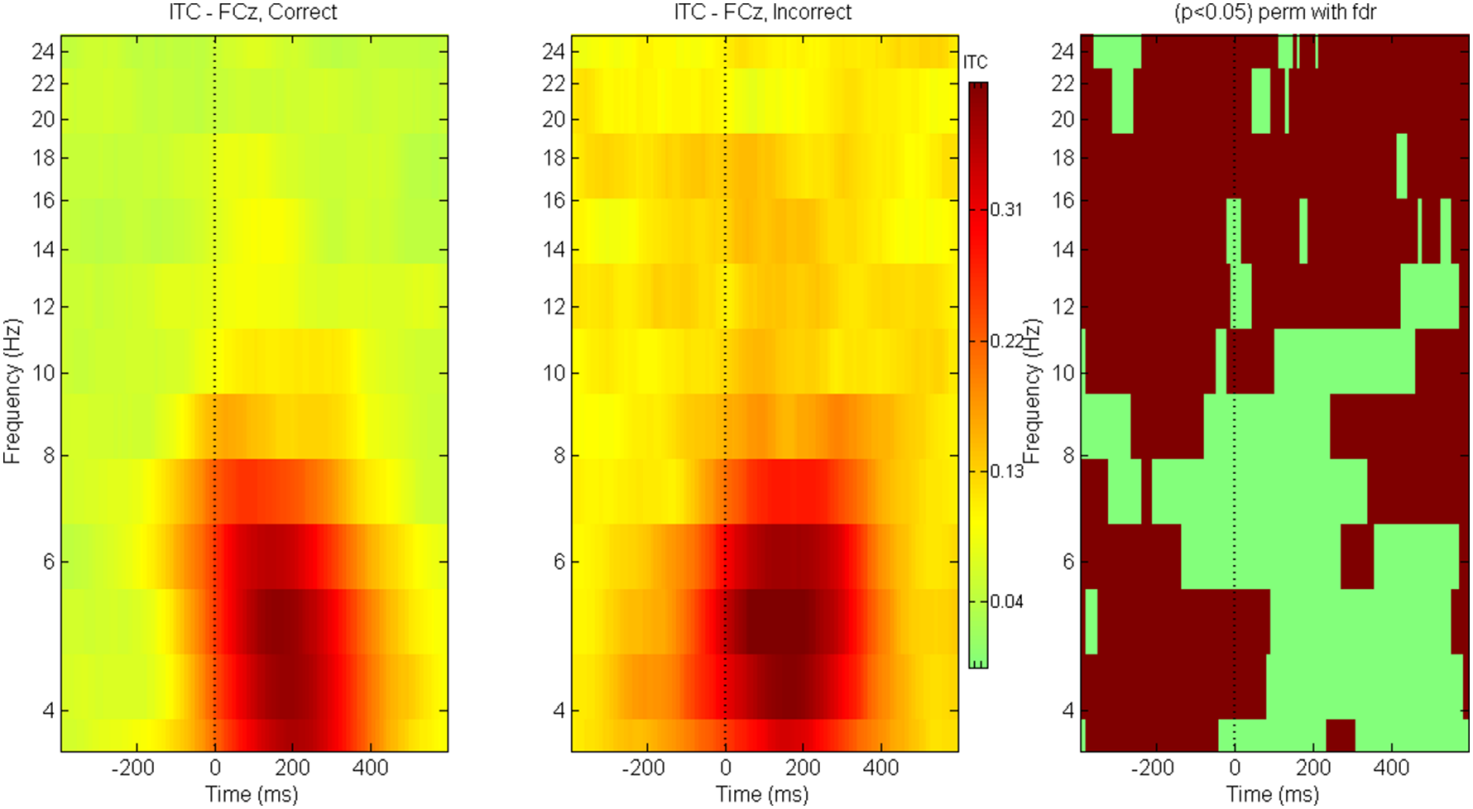
**ITC for ERN**. Inter trial coherence for ERN at electrode FCz for game level *Discern*. Panel on the right indicates the regions with significant differences across conditions (*p* < 0.05 Paired *t*-test with 2000 permutations and FDR correction).

**Figure 13:**
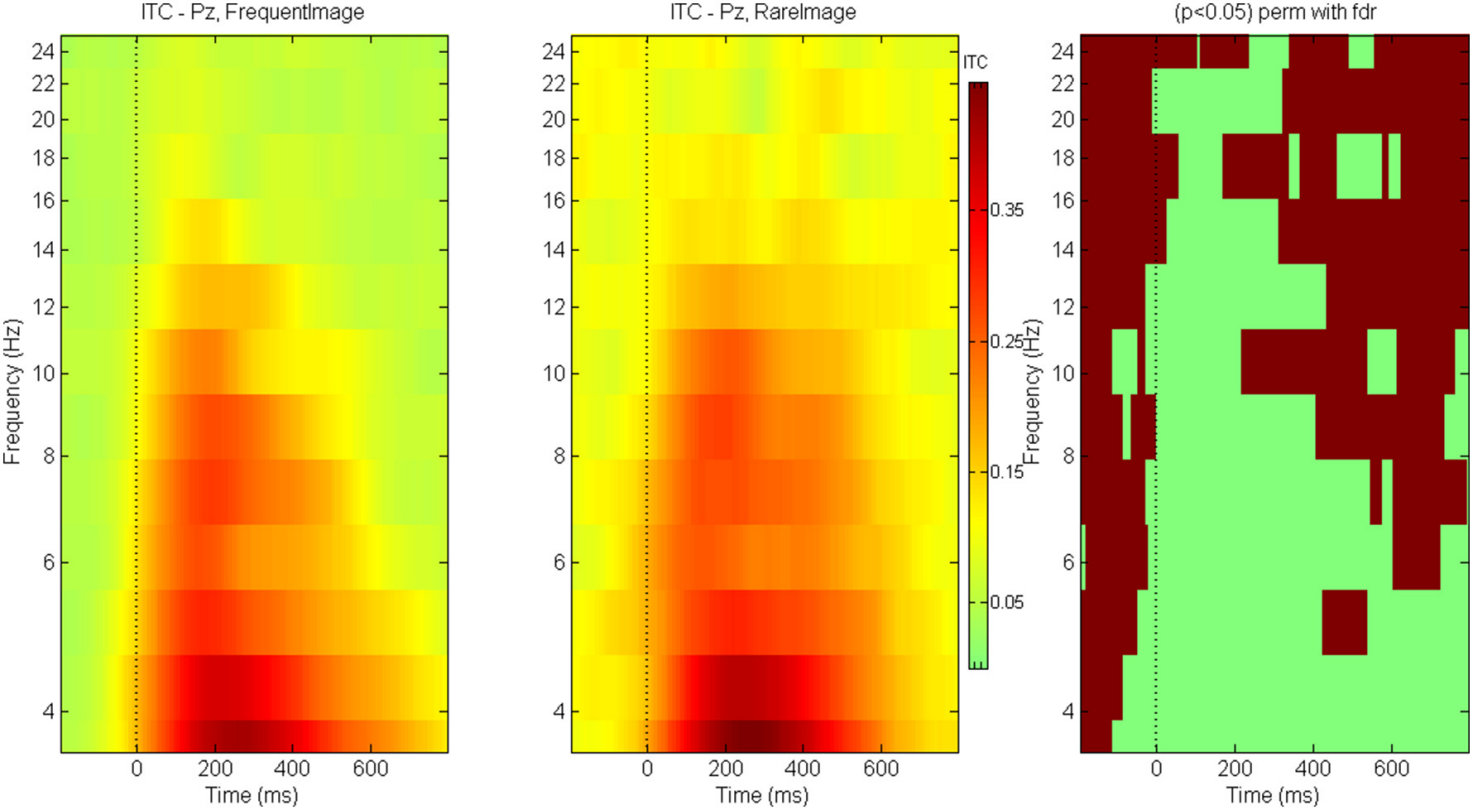
**ITC for P300**. Inter trial coherence for P300 for game level *Who*. Panel on the right indicates the regions with significant differences across conditions (*p* < 0.05 Paired *t*-test with 2000 permutations and FDR correction).

Between-condition ITC differences are shown for ERN (Figure 12) and P300 (Figure 13) components. The brown colored areas on the rightmost panel show the regions with significant differences between the conditions (*p* < 0.05, using robust measure of paired *t*-test; computed with 2000 permutations and FDR correction for multiple comparisons). The ITC for ERN at electrode FCz is globally enhanced for trials with *Incorrect* responses as compared to those with *Correct* responses. The response locked ITC is similar across both kinds of trials in the theta-lower alpha frequency range after about 100 ms post response.

For P300 at electrode Pz, trials with *Rare* stimuli have greater inter-trial coherence (as compared to trials with *Frequent* stimuli) at baseline and at all frequencies except during 0–600 ms post stimulus where the trials are equally coherent for both types of stimuli.

### Measure projection analysis (MPA)

**Figure 14:**
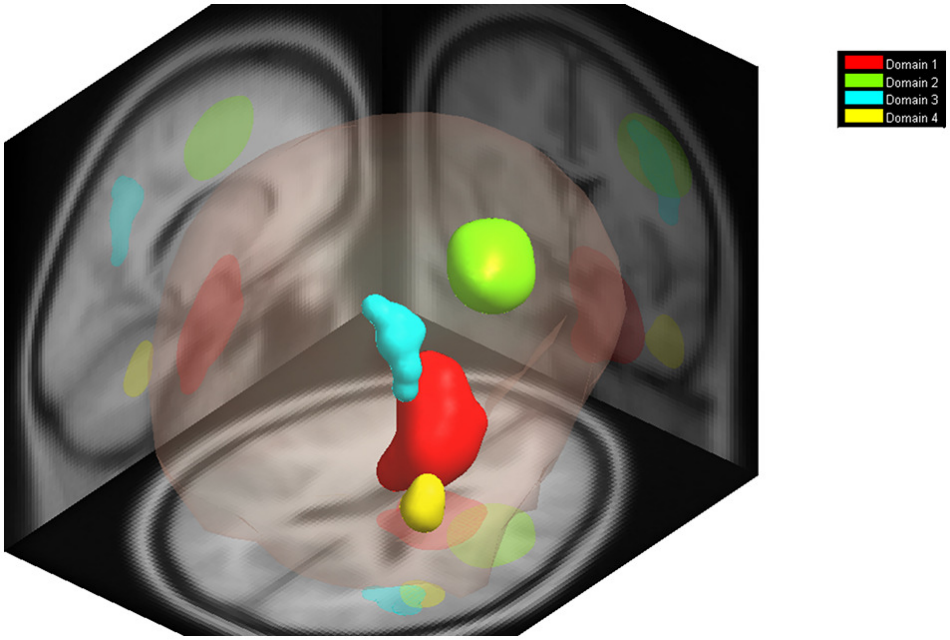
**Measure Project Analysis for P300**. Measure Projection for localizing component dipoles into four significant domains for the P300 ERP measure [*p* < 0.01 Paired *t*-test (right-tailed) with 2000 permutations—default settings]. The prominent anatomical regions include the Right Hippocampus, Right Precentral Gyrus, Right Angular Gyrus, and the Cerebellum.

MPA for P300 yielded four domains as shown in Figure 14. The prominent anatomical regions included the Right Hippocampus, Right Precentral Gyrus, Right Angular Gyrus, and the Cerebellum.

### Quantitative comparisons with previous ERP studies

Table 1 shows amplitude effect sizes (Cohen's d calculated as compared to baseline) of C1, N170, ERN, and P300 from our study and representative results from existing literature focused on these particular ERPs. While our study is able to simultaneously elicit more ERPs than any other approach, the amplitude effect sizes are comparable to those in studies that assess one or two ERPs at a time.

**Table 1:** **Quantitative comparison of sample ERPs with existing literature**.

	**Our study**	**Other studies**
Number of ERPs assessed	10	Four: Kappenman and Luck, 2012, typically One or Two
**Sample ERP**	**Effect sizes of example ERP components**	
C1	−1.0±0.44 μV	−3.2±0.4 μV
	*N* = 18	*N* = 18
	(Cohen's *d* = 2.27)	(Cohen's *d* = 8), Fu et al., 2010
N170	3.43 ± 2.38 μV	−7.85±1.43 μV
	*N* = 18	*N* = 14
	(Cohen's *d* = 1.44)	(Cohen's *d* = 1.73), Rossion and Caharel, 2011
ERN	−2.3±1.42 μV	−3.65±2.10 μV
	*N* = 18	*N* = 40
	(Cohen's *d* = 1.62)	(Cohen's *d* = 1.74), Hajcak and Simons, 2008
P300	3.23 ± 1.49 μV	10.75 ± 4.63 μV
	*N* = 18	*N* = 15
	(Cohen's *d* = 2.17)	(Cohen's *d* = 2.32), Shaikh et al., 2013

### Qualitative feedback from participants

The qualitative feedback from the gamified approach has been compelling. All subjects during the study and subsequent acquisitions (over 200 acquisitions done so far for various ongoing studies in our lab) have liked the ANGEL paradigm. Sample feedback: “I enjoyed it”; “I have learnt a lot about myself”; “My concentration is good but I found that I can improve”; “Awesome!”; “Great new experience”; “The last level was a little difficult, but fun”. So far, we have had only one mixed feedback from a subject who said: “I feel sleepy when I concentrate.” This subject was part of a different ongoing study and his acquisition had to be stopped at the third level.

## Discussion

The present study validated the usefulness of the ANGEL paradigm design for simultaneous recording of multiple ERPs and the multiple analysis possibilities. Our study demonstrates that the ANGEL approach is able to simultaneously elicit multiple ERPs in a significantly reduced time frame, elicit the chosen ERPs in a reliable manner and is also ecologically valid as it is more representative of real-world task performance in the presence of multiple audio-visual distracters. The ERP waveform morphology is as expected with statistically significant between-condition differences seen in the various measures.

The ANGEL design introduces a number of gamification elements to the typical oddball paradigm. These are—clear rules that change with context, variety of stimuli, progressive complexity, regular feedback for performance monitoring and improvement, achievable but challenging self-driven goals. Specifically:

1. The task provides variety in terms of rules that bring changes at each level. The task starts with alternating blocks requiring response inhibition (discrimination with passive observation) and speeded up response in the presence of mild distractors, then the number of distractors is increased and finally the rules of the game are changed twice requiring complex discrimination and rapid decision making.
2. To sustain interest, a variety of stimuli are presented in an unpredictable manner (the oddball experience is at an abstract/category level rather than with a finite/concrete set of known images).
3. The visual stimuli are two-tone images that require gestalt perception and so add to the level of challenge at the final level especially when “*FaceAbsent*” stimuli are presented.
4. The complexity levels are scaled to generate and maintain motivation. The easiest level is fast paced but easy so as to provide high initial motivation. The toughest level is quite challenging and yet the rules are simple so the participants stay engaged and feel that they can perform well if they try hard.
5. We provide quantitative speed and accuracy feedback along with messages that encourage better performance in terms of either accuracy or speed depending on the level of accuracy.

The ERPs waveforms obtained by ANGEL are in line with the existing literature—(Luck and Kappenman, 2012) provides a comprehensive review of several of the neurocognitive variables that can be studied using ANGEL. The paradigm simultaneously assesses multiple neural processes such as: auditory and visual perception, attention, set shifting, memory, discrimination, corollary discharge, motor readiness, conflict processing, and multiple levels of decision making under changing rules.

While collecting reaction time and accuracy for providing feedback and as behavioral measures, the ERPs (and associated cognitive parameters) that are assessed are: C1 for early visual processing (Kelly et al., 2008), P50 for early auditory processing (Smith et al., 2013), MMN for discriminative auditory processing (Chen et al., 2014), the N1-P2 complex as a marker of corollary discharge—for self-inference or agency detection (Wang et al., 2014), N170 for recognition and differential object perception (Caharel et al., 2013), N2pc for covert attention shift (Cespón et al., 2013), LRP for motor readiness (Vainio et al., 2014), P300 for sustained attention, working memory, context updating and discrimination (Shaikh et al., 2013), and finally ERN for self-regulation and performance monitoring (Arbel and Donchin, 2014). We discuss the results for four of these ERPs in more detail below:

In ANGEL, the C1 wave is generated by visual distracters (unattended stimuli)—the stimulus in the upper visual field generates a negative potential and the stimulus in the lower visual field elicits a positive potential which is often indistinguishable from P1 as expected. The peak amplitude is quite small as is expected from early visual processing of unattended stimuli (Kelly et al., 2008) lending support to the hypothesis that attention modulates early visual processing.

The N170 was generated by covert detection of patterns (faces and shapes and their distorted forms). The overall amplitude for covert shape detection was greater than for covert face detection. This is in contrast with the N170 face-effect that is cited in the literature (See Caharel et al., 2013 and its references). We attribute this difference to the fact that our stimuli require gestalt perception of two-tone images rather than simple and clear images. Additionally, most of our subjects reported that Kanizsa triangles are much easier to perceive rapidly than Mooney faces. The N170 waveform in our study has its greatest negative peak close to 220 ms rather than at 170 ms. The waveform morphology is similar to that in the literature and is consistent and complexity dependent across different game levels which demonstrates the reliability and shows that face/object detection is modulated by task complexity. However, the reason for the delay needs to be further explored.

The ANGEL game provides scaled complexity levels. The first two levels are simple and errors committed are infrequent. However, the third level, *Discern and Decide*, is highly complex and participants commit more errors as evident by the larger ERN response for incorrect responses (Arbel and Donchin, 2014). *Incorrect* trials show enhanced inter trial coherence across all observed frequencies during the baseline (prior to button response) as compared to the *Correct* trials which is a remarkable indicator of greater cerebral resource allocation during conflicts or uncertain situations.

In ANGEL, an abstract version of the oddball paradigm has been used with different *Standard* and *Rare* stimulus types presented for each block. We find that the P300 waveform is elicited at the single block levels even when the type of the *Standard* stimuli changes in every block. The attention and working memory related aspects are seen via the theta enhancement and concomitant alpha-beta suppression. Interestingly, for *Rare* stimuli, we see a significant global rise in baseline inter-trial coherence (i.e., are phase-locked) across all observed frequencies. This could be due to the expectancy effect that builds up in the oddball paradigm. We have also observed right hemisphere dominance in the P300 ERP sources. The gamified version of a typical oddball paradigm of the ANGEL appears to be engaging. Keeping the “game” simple enables simultaneous study of multiple canonical ERPs without introducing confounds. Providing speed and accuracy scores along with motivational feedback after every two blocks avoids confounds of boredom and fatigue.

In ANGEL, we assess more simultaneous ERP components than any other known approach used in ERP studies (Table 1). While larger effect sizes are better, quantitative evaluation of amplitude effect sizes reveal that the effect sizes in our study are comparable to other studies that are focused on the ERP in isolation. For C1, the effect size in our study is much lesser than the referenced paper but it is a good effect size in itself and the difference is most probably due to the fact that we used distracter (non-attended) images to elicit this ERP.

We have not needed to adapt the ANGEL paradigm to suit different populations. Subjects with widely differing backgrounds have been able to perform the task and enjoy it. Examples of diversity include: *Mental health*: patients with schizophrenia, healthy subjects, meditators; *Education*: low/no literacy levels to PhDs; *Age*: from twenties to late seventies; *Computer familiarity*: computer naïve to professional programmers.

We believe patients with neurological diseases may also be able to perform the task depending on their level of sensorimotor difficulties. In the various ongoing studies in our lab, the participant with the lowest WAIS III (Wechsler Adult Intelligence Scale for Matrix Reasoning) score (8), was also able to complete the task. Those with greater cognitive difficulties may be able to perform the first two levels of the task—but this needs to be assessed.

Participants found their performance scores improving during the first two levels with almost everyone being able to achieve 100% accuracy for at least one block and many people able to regularly achieve full accuracy while reducing reaction times. These two levels provide participants with an opportunity for steady improvement in their performance both in terms of accuracy and speed of response in spite of visual and auditory distractors. This helped in maintaining high motivation levels to start the complex third level of task. The third level is more challenging and we find that participants make more errors and take additional time to respond. Having such scaled complexity levels allows sufficient number of trials with correct and incorrect responses.

Apart from the obvious benefits of studying multiple ERPs simultaneously, one can study other cognitive functions using the ANGEL approach. We can evaluate the effects of changing individual elements of the game such as difference between passive observation vs. active response, presence vs. absence of response locked distractors (to study the corollary discharge mechanism), and the set-shifting ability when rules are changed while performing a challenging task. The power of such multilevel gamified approach is that we can examine each ERP at different levels of complexity and examine changes if any across levels. The results of N170 changes across the levels of the game provide a counterintuitive example of how game complexity impacts covert pattern detection. The impact of cognitive loading on other ERPs can similarly be examined.

Importantly, the design provides the flexibility to analyze the data under many different conditions that may not have been originally planned for the study or may be required based on initial analysis. For example, one can study the error related negativity (ERN) averaged from all rare trials that elicited an incorrect response to a meaningful gestalt stimulus.

The ANGEL approach has already been used in multiple studies in our lab with several hundred participants across different study populations, with multiple acquisition systems, and multiple acquisition domains like ERP and fMRI. While the ANGEL paradigm allows us to study many ERPs simultaneously, several ERPs of interest remain unexplored—for example, we might be interested in the N400 to study semantic processing (Duncan et al., 2009). It is possible to look at experimentally manipulating additional neurocognitive variables or to replace some of the existing variables with new ones. This, however, needs a lot of care to avoid the introduction of confounds.

We freely provide the source code (https://github.com/nphynimhans/ANGEL) for our design and software to the community so that others can use it to study different populations of interest and adapt or extend the code according to their research interests. We anticipate that a gamified approach to assessing neurocognition can be a big step forward for our research field.

## Conclusion

This paper on the ANGEL approach describes the paradigm design and demonstrates its validity and utility in the study of multiple neurocognitive variables such as auditory and visual perception, attention, set shifting, memory, discrimination, corollary discharge, motor readiness, conflict processing, and multiple levels of decision making under changing circumstances.

The assessment of neurocognition with the ANGEL approach: is ecologically more valid (realistic sensory-motor activity); intrinsically motivating (game, performance reward); highly efficient (multifold reduction in overall acquisition time) considering the number of variables assessed; and having high statistical power (increased number of trials and decrease in possible confounds).

ANGEL provides an opportunity to evaluate influences (isolate main and interaction effects of different variables) and provides spectrum coverage (whole brain neurocognition study) while being user friendly (for subject and investigator) and automatable (over 500 event markers) to allow focusing on the relevant area of interest with desired extent of granularity.

Thus, the ANGEL framework allows simultaneous assessment of several ERPs providing an opportunity for a more holistic neurocognitive study.

## Author contributions

AN, AS, and BK conceptualized the study; AN carried out the data acquisition and analysis; AN and AS designed the task; AN and AS wrote the data analysis scripts; BK, JJ, and SM critically evaluated the study; all authors contributed to writing the manuscript.

### Conflict of interest statement

The authors declare that the research was conducted in the absence of any commercial or financial relationships that could be construed as a potential conflict of interest.
